# Phosphatidic acid is involved in regulation of autophagy in neurons in vitro and in vivo

**DOI:** 10.1007/s00424-024-03026-8

**Published:** 2024-10-08

**Authors:** Maximilian Schiller, Gregory C. Wilson, Simone Keitsch, Matthias Soddemann, Barbara Wilker, Michael J. Edwards, Norbert Scherbaum, Erich Gulbins

**Affiliations:** 1 LVR-University Hospital Essen, Department of Psychiatry and Psychotherapy, Faculty of Medicine, Faculty of Medicine, University of Duisburg-Essen, 45147 Essen, Germany; 2 Department of Surgery, University of Cincinnati College of Medicine, Cincinnati, OH 45267-0558 USA; 3 Department of Molecular Biology, University Hospital Essen, University of Duisburg-Essen, Hufelandstrasse 55, 45122 Essen, Germany

**Keywords:** Major depression, Phosphatidic acid, Ceramide, Autophagy, Gangliosides

## Abstract

Major depressive disorder (MDD) is a common and severe psychiatric disease, which does not only lead to variety of neuropsychiatric symptoms, but unfortunately in a relatively large proportion of cases also to suicide. The pathogenesis of MDD still requires definition. We have previously shown that ceramide is increased in the blood plasma of patients with MDD. In mouse models of MDD, which are induced by treatment with corticosterone or application of chronic unpredictable stress, increased blood plasma ceramide also increased and caused an inhibition of phospholipase D in endothelial cells of the hippocampus and reduced phosphatidic acid levels in the hippocampus. Here, we demonstrated that corticosterone treatment of PC12 cells resulted in reduced cellular autophagy, which is corrected by treatment with phosphatidic acid. In vivo, treatment of mice with corticosterone or chronic unpredictable stress also reduced autophagy in hippocampus neurons. Autophagy was normalized upon i.v. injection of phosphatidic acid in these mouse models of MDD. In an attempt to identify targets of phosphatidic acid in neurons, we demonstrated that corticosterone reduced levels of the ganglioside GM1 in PC-12 cells and the hippocampus of mice, which were normalized by treatment of cells or i.v. injection of mice with phosphatidic acid. GM1 application also normalized autophagy in cultured neurons. Phosphatidic acid and GM1 corrected stress-induced alterations in behavior, i.e., mainly anxiety and anhedonia, in experimental MDD in mice. Our data suggest that phosphatidic acid may regulate via GM1 autophagy in neurons.

## Introduction

Major depressive disorder (MDD) is a severe and chronic disease with a lifetime prevalence of more than 10% [2, 13, 16]. MDD is also often a life-threatening or even fatal illness since approximately 10% of MDD patients attempt suicide [2]. Depressed mood, lack of drive, and anhedonia are the main symptom of MDD, but patients also suffer from the feeling to be dead inside, fear and anxiety, feelings of worthlessness, insomnia, and concentration deficits [2].

Classic antidepressants are most often used to treat MDD, but these drug treatments fail in approximately one third of the cases requiring treatment with a second antidepressant or another therapy modality [2, 11, 63]. More importantly, classic antidepressants such as fluoxetine, imipramine, amitriptyline, or escitalopram show a delayed onset of action and require 2–3 weeks to exhibit a therapeutic benefit [2, 11, 63].

Electroconvulsive therapy is a fast-acting and relatively reliable treatment option for MDD [15, 59]. However, electroconvulsive therapy is usually only applied to severe cases and for a limited time since it requires general anesthesia, which cannot be applied unlimited times [15, 59]. Other treatment options such as sleep deprivation or transcranial magnetic stimulation can be also used for treatment of MDD [2, 57], but their long-term therapeutic effects seem to be limited [27, 57]. Ketamine that targets *N*-methyl-d-aspartate (NMDA) receptors has the advantage of a fast action against MDD [3, 40, 55, 59, 60, 65], but addiction, dissociation, altered feelings, abuse, and lack of efficacy in a significant proportion of patients are major problems [3, 11, 40].

The pathogenesis of MDD still requires definition despite its devastating impact on diseased individuals. Originally, it was suggested that MDD is caused by alterations of neurotransmitter functions and concentrations in the synaptic space, consistent with the notion that many classical antidepressants inhibit the uptake of monoamine and other neurotransmitters resulting in increased extracellular concentration and enhanced receptor binding [9, 32, 39, 63]. However, some of the drugs, for instance, tianeptine, even promote the uptake of neurotransmitters questioning the monoamine concept of MDD [1]. Also, pharmacological depletion of monoamines failed to induce depression in healthy people [9, 10]. Reduced proliferation of hippocampus neurons was also suggested as concept for pathogenesis [44, 50, 61], but inhibition of neuronal proliferation does not necessarily result in MDD [50]. Furthermore, the fast action of drugs such as ketamine or of electroconvulsive therapy [3, 40, 60] questions the hypothesis of a reduced neuronal proliferation as cause for MDD. Furthermore, it was suggested that MDD is caused by aberrant neuroplasticity and alterations in growth factors and their receptors [16, 39, 46, 49], but the clinical relevance of these findings is unknown. In addition, alterations of (pro-inflammatory) cytokines in the blood or brain were suggested to be involved in MDD [e.g., 37, 43]. A more recently proposed concept suggested a distinct dysfunction of endothelial cells and the blood–brain barrier in the brain, in particular in the expression of claudin 5 in brain endothelial cells [45, 64]. Furthermore, it was demonstrated (i) that stress induces a reduction of autophagy in hippocampus neurons, at least in stressed mice, (ii) that at least some antidepressants such as amitriptyline or fluoxetine improve autophagy in experimental mouse models of MDD, and (iii) that the antidepressive effect of these drugs in these experimental models of MDD depends on the induction of autophagy [19, 21, 66]. However, the in vivo relevance of reduced autophagy for MDD in humans is unknown. Some of these factors may act in concert, for instance, an increase of cytokines in the blood, an altered blood–brain barrier, decreased autophagy, and reduced neuronal proliferation. It might be also possible that many pathogenetic factors finally result in similar responses of the central nervous system and development of clinical MDD symptoms. However, it is also possible that many of these mechanisms are downstream of other, unknown pathogenetic factors.

Several studies revealed that ceramide accumulates in the blood plasma of human individuals with MDD and in mice upon application of several forms of stress in mice [4, 33, 52]. The patients in the study by Schumacher et al. were treatment naïve suggesting that ceramide may function as an early pathogenetic factor of MDD [52]. Ceramide can be hydrolyzed from sphingomyelin by acid, neutral, and alkaline sphingomyelinases [22, 25, 51]. Ceramide can be also generated de novo by the ceramide synthase pathway [18, 47]. We demonstrated that the increase of blood plasma ceramide in animal models of corticosterone- or chronic unpredictable stress–induced MDD is mediated by neutral sphingomyelinase 2 since mice deficient for this enzyme were protected from stress-induced MDD [53]. Currently, it is unknown how the neutral sphingomyelinase 2 is activated by corticosterone or chronic unpredictable stress and how the enzyme is involved in the induction of experimental animal models of MDD. We further showed that intravenous injection of anti-ceramide antibodies or neutral ceramidase that either neutralized or consumed blood plasma ceramide rapidly, i.e., within hours, abrogated stress-induced MDD [42].

Mechanistically, we proposed that blood plasma ceramide inhibits phospholipase D in endothelial cells of the brain, in particular the hippocampus. Inhibition of phospholipase D in endothelial cells and possibly also other cells resulted in a reduced concentration of phosphatidic acid in the hippocampus [52]. The effects of ceramide on other cells still need to be defined. The reduced concentration of phosphatidic acid in the hippocampus may result in symptoms of MDD and, in accordance, i.v. injection of phosphatidic acid into experimentally depressed mice or treatment of MDD patients with phosphatidic acid rapidly improved MDD [29, 30, 52].

This novel concept indicates that MDD is initiated as a peripheral response to stress that evolves to manifest itself in the brain by a reduced formation of phosphatidic acid that seems to be required for normal function of neurons [14, 52].

Phosphatidic acid consists of the glycerol backbone which is esterified with two fatty acids usually at position 1 and 2 (sn-1 and sn-2) and a phosphate usually at position 3 (sn-3). However, the exact composition of phosphatidic acid altered in the brain of mice with experimental MDD is currently unknown.

Targets of phosphatidic acid in neurons are largely unknown and, thus, we aimed to identify downstream targets of phosphatidic acid in the hippocampus. We demonstrate that phosphatidic acid corrects stress-induced inhibition of autophagy in mice with experimental MDD. The effect of phosphatidic acid on autophagy seems to be mediated by a regulation of the expression of the ganglioside GM1 by phosphatidic acid.

## Material and methods

### Mice and treatments

We used C57BL/J6 mice at an age of 8–12 weeks. We used 6 mice per group. Corticosterone (Sigma) was administered at 100 mg/L in the drinking water for 6 days. Phosphatidic acid was injected i.v. twice daily on day 5 after initiation of corticosterone treatment, i.e., 24 and 12 h prior to analysis of the mice. We injected 4 μg/g body weight phosphatidic acid suspension into the tail vein. We used male and female mice. All studies were performed in accordance with animal permissions of the IACUC Cincinnati.

### PC-12 cells

PC-12 cells were grown in MEM, supplemented with 10% horse serum, 10 mM HEPES (pH 7.3), 10 mM penicillin/streptomycin, 1 mM sodium pyruvate, and 2 mM L-glutamine. The cells were incubated for 4 days with 0.5 μg/mL corticosterone, 0.5 μg/mL corticosterone + 5 μM phosphatidic acid, 0.5 μg/mL corticosterone + 10 μM GM1, or left untreated. Cells were then washed twice in HEPES/saline (H/S; 132 mM NaCl, 20 mM HEPES [pH 7.4], 5 mM KCl, 1 mM CaCl_2_, 0.7 mM MgCl_2_, 0.8 mM MgSO_4_); lysed for 5 min on ice in 3% NP40, 0.1% Triton X-100, 25 mM HEPES, 10 mM EDTA, 10 mM sodium pyrophosphate, 10 mM sodium fluoride, 125 mM NaCl, and 10 µg/mL aprotinin/leupeptin; centrifuged for 5 min at 14,000 rpm; and the supernatants transferred into 5 × SDS-sample buffer. Samples were then incubated for 5 min at 95 °C and used for western blotting as described below.

### Western blot analysis of phospho-Ulk, phospho-beclin, phospho-PI3-K/Vps34, and p62 in the hippocampus or PC-12 cells

Mice were sacrificed; the hippocampus was prepared and removed, shock frozen, and homogenized with a tip sonicator in 300 μL of 3% NP40, 0.1% Triton X-100, 25 mM HEPES, 10 mM EDTA, 10 mM sodium pyrophosphate, 10 mM sodium fluoride, 125 mM NaCl, and 10 μg/mL aprotinin/leupeptin; lysed for 5 min at 4 °C; centrifuged at 14,000 rpm for 5 min at 4 °C; the supernatants added to 5 × SDS-Laemmli buffer; and finally incubated at 95 °C for 5 min. Samples were separated by 7.5% or 10% sodium dodecyl sulfate–polyacrylamide gel electrophoresis (SDS-PAGE). Proteins were electrophoretically transferred onto nitrocellulose membranes, blocked in Starting Block (TBS) blocking buffer (Thermo Fisher Scientific, #37542) for 60 min, washed twice in TBS/0.05% Tween, and incubated for 60 min at room temperature with antibodies specific for phospho-Ulk serine 555 (Cell Signaling, #5869), phospho-Ulk serine 757 (Cell Signaling, #6888), phospho-Beclin (Cell Signaling, #84966), and phospho-PI3-K/Vps34 (Cell Signaling, #13857) or p62 (Sigma, #P0067). All antibodies were diluted 1:1000-fold in Starting Block (TBS) blocking buffer. Blots were then washed six times, 10 min each in TBS/0.05% Tween, incubated for 30 min with alkaline phosphatase (AP)–coupled antibodies directed against the primary antibodies, washed again six times in TBS/0.05% Tween, twice in alkaline wash buffer, and developed using the CDP-STAR with NitroBlockII Enhancer system (Perkin Elmer).

### Transfections of RFP-62, RFP-Lc3b, and RFP-GFP-Lc3b

The transfections were performed as previously described [19]. PC-12 cells were grown on glass cover slips in 24-well plates for microscopy studies or in 12-well plates in suspension for flow cytometry. The cells were treated with corticosterone (0.5 μg/mL) for 4 days in the presence or absence of 5 μM phosphatidic acid, or left untreated. The cells were then infected with a Baculovirus system containing a mammalian promoter (Invitrogen, #P36236, 36239, 36241). To this end, the cells were infected with the constructs at a MOI of 30 viral particles encoding RFP-p62 or RFP-Lc3B per cell and cells were analyzed after 24 h. We determined the number of red dots per cell in 50 cells per sample from six independent transfections (total of 300 cells) to measure p62 and Lc3B at vesicular structures. The fusion of autophagosomes with lysosomes, i.e., the formation of autophagolysosomes, was studied by transfection of PC-12 cells with a baculovirus tandem sensor RFP-GFP-Lc3B (Invitrogen) at a MOI of 30 viral particles per cell. Since the fluorescence of GFP is pH sensitive and quenched upon acidification, while the fluorescence of RFP is pH insensitive, the percentage of phagolysosomes with a high RFP-fluorescence and low GFP-fluorescence versus total phagosomes with high RFP- and GFP-fluorescence can be analyzed by flow cytometry.

### Immunohistochemical studies

Mice were stressed with corticosterone to induce MDD-like symptoms and treated with phosphatidic acid or left untreated. Mice that were completely left untreated served as controls. Mice were euthanized and immediately perfused via the left heart for 2 min with 0.9% NaCl followed by a perfusion with 4% paraformaldehyde (PFA) buffered in PBS (pH 7.3) for 15 min. Brains were removed, fixed for an additional 36 h in 4% buffered PFA in PBS, embedded in paraffin, sectioned at 6 μm, and dewaxed. To expose antigens, sections were then incubated for 30 min with pepsin (Digest All; Invitrogen, Darmstadt, Germany) at 37 °C, washed three times in PBS, and blocked for 10 min with PBS, 0.05% Tween 20, and 5% fetal calf serum (FCS). The samples were washed again in H/S and then immunostained for 45 min with anti-phospho-Ulk serine 555, anti-phospho-Ulk serine 757, anti-phospho-Beclin, anti-phospho-PI3-K/Vps34, or anti-p62 antibodies. All antibodies were diluted 1:200 in H/S supplemented with 1% FCS. The samples were then washed three times, each 5 min, in PBS supplemented with 0.05% Tween 20, once in PBS, incubated for 45 min with Cy3-coupled donkey anti-rabbit IgG F(ab)_2_ fragments (1:500; Jackson ImmunoResearch, Newmarket, UK), and washed again three times, 5 min each, in PBS supplemented with 0.05% Tween 20 and once in PBS. Finally, the sections were embedded in Mowiol and analyzed on a LEICA TCS SL confocal microscope using a 40 × lens (400-fold magnification). Control stainings with isotype-matched control antibodies showed very weak or no signals and served as specificity controls.

### Measurement of GM1 in vitro

PC-12 cells were labeled with 1 μCi/mL [^14^C]galactose for 48 h followed by treatment with 0.5 μg/mL corticosterone for 48 h. We then extracted the cells into CHCl_3_:CH_3_OH:H_2_O (1:2:0.8, v/v/v), separated the phases by 5-min centrifugation at 14,000 rpm, collected and dried the lower phase, separated gangliosides on TLC silica 60 plates (Merck #1.05748.0001) using CHCl_3_:CH_3_OH:0.22% CaCl_2_ (60:35:8, v/v/v), and quantified GM1 employing a Fuji-Imager. GM1 was identified by co-migration with a standard. Samples were normalized for protein determined by a Bradford assay and cell number.

### Measurement of GM1 in the hippocampus after in vivo treatment

Mice were left untreated, treated with corticosterone at a concentration of 0.1 mg/mL in the drinking water for 5 days, or treated with corticosterone and i.v. injected twice with 4 μg/g body weight phosphatidic acid suspension at day 4, i.e., 24 and 12 h prior to further analysis. The animals were sacrificed and the hippocampus was removed, homogenized by sonication in water, extracted in CHCl_3_:CH_3_OH:H_2_P (1:2:0.8, v/v/v), and lipids were analyzed as above. The TLC plates were sprayed with orcinol reagent, dried, developed at 120 °C for 30 min, and analyzed by densitometry. GM1 was quantified by comparison with a standard curve of known GM1 amounts. Protein was determined by a modified Bradford assay (BioRad).

### Phosphatidic acid measurements

Phosphatidic acid was measured in blood plasma and cell culture medium following the protocol of the vendor (PromoKine, #PK-CA577-K748). Aliquots (200 μL) were extracted in 750 μL CHCl_3_:CH_3_OH:12N HCl (2:4:0.1, v:v:v) and each 250 μL CHCl_3_ and 1 M NaCl, vortexed, and the organic phase was dried in a speed vac. Dried samples were dissolved in 5% Triton X-100 and the enzymatic reaction was started by addition of an enzyme mix that hydrolyzes phosphatidic acid to an intermediate that is converted to a fluorescence substrate. Fluorescence was analyzed at excitation/emission of 535/587 nm, and concentrations were determined using a standard curve.

### Behavioral studies

Behavioral testing was performed between 8:00 a.m. and 10:00 a.m. under diffuse indirect room light. Novelty-suppressed feeding test: Mice were fasted for 24 h and placed in a new cage with a piece of food on a piece of white paper in the middle of the arena. The time was recorded during which the mice explored the new environment before they began eating. Light/dark box test: Mice were placed in a dark and safe compartment that was connected via a 5 × 5-cm aperture with rounded-down corners with an illuminated, open, and thus aversive area. The times the mouse spent in the different compartments were recorded. Coat state test: The appearance of the coat (groomed vs. unkempt coat) was scored on the head, neck, back, and ventrum with either a 0 for a normal status or 1 for an unkempt status. Splash test: We spotted 200 µL of a 10% sucrose solution onto the mouse’s snout, and the latency to begin grooming and the grooming frequency over 5 min were measured. Forced swim test: Mice were placed in a glass beaker filled with water (21–23 °C) for 15 min. After 24 h, the mice were again placed in a water-filled beaker for 6 min, and the time of immobility during the last 4 min was recorded. Mice were judged immobile when they moved only to keep their heads above water.

### Statistical analysis

Data are expressed as arithmetic means ± SD. We employed one-way ANOVA followed by post hoc Student's *t*-tests for all pairwise comparisons of continuous variables from independent groups. All values were tested for normal distribution. We applied Bonferroni correction for multiple testing prior to calculating *p* values for the pairwise comparisons. A *p* value of 0.05 or less (two-tailed) was considered indicative of statistical significance. The sample size planning for the continuous variables in vivo infection experiments was based on two-sided Wilcoxon–Mann–Whitney tests (free software: G*Power Version 3.1.7 of the University of Dusseldorf, Germany). Investigators were blinded for histology experiments and animal identity. Western blots and confocal fluorescence microscopy stainings were quantified using ImageJ and are expressed as arbitrary units (a.u.).

## Results

**Fig. 1:**
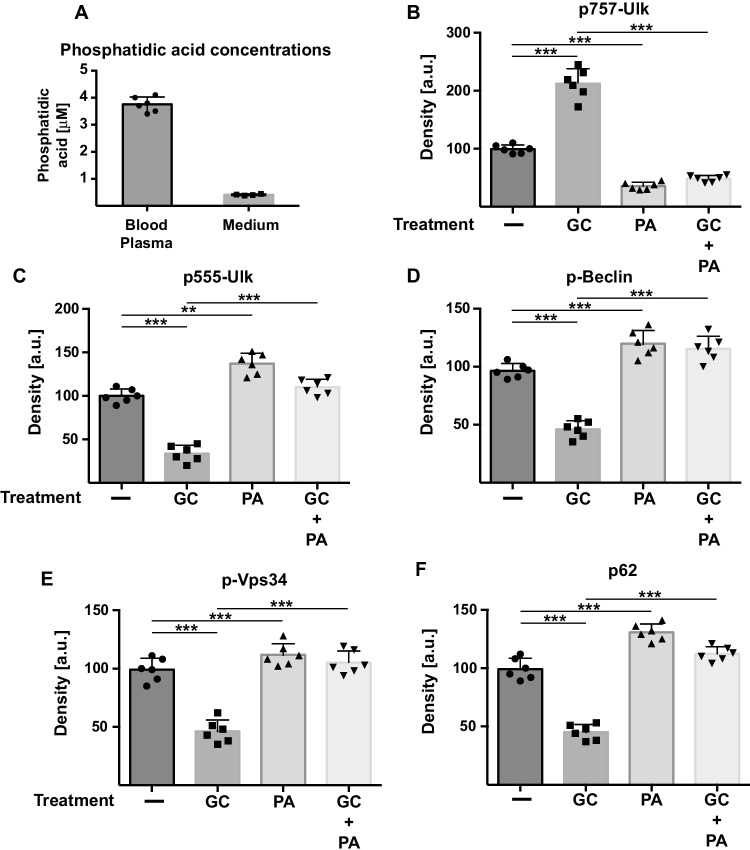
Phosphatidic acid promotes autophagy in corticosterone-treated neurons. (**A**) Concentrations of phosphatidic acid were determined in blood plasma and in MEM medium containing 10% fetal calf serum using a commercial ELISA. (**B–F**) PC-12 cells were stressed with 0.5 μg/mL corticosterone (GC) or left untreated in the presence or absence of 5 μM phosphatidic acid (PA). Stress increased the inhibitory phosphorylation of Ulk (S757) (**B**) and reduced the activating phosphorylation of Ulk (S555) (**C**), Beclin (**D**), PI3-K/Vps34 (**E**), and the expression of p62 (**F**). Phosphatidic acid treatment of the cells reversed the effects on the regulators of autophagy (**B–F**). Shown are the quantitative analyses of western blots obtained from PC-12 extracts (**B–F**). The western blots were quantified by densitometry; *n* = 4 in (**A**), *n* = 6 in (**B**–**F**); given is the mean ± SD. **p < 0.01, ***p < 0.001, ANOVA and post hoc Tukey test

To test whether phosphatidic acid is involved in the regulation of autophagy, we determined the concentration of phosphatidic acid in blood plasma of mice and in cell culture medium. These studies revealed a concentration of phosphatidic acid of 3.75 ± 0.27 μM in blood plasma of healthy mice (Fig. 1A). This concentration is similar to that in cell culture medium containing 10% fetal calf serum of approximately 0.41 ± 0.03 μM. The concentrations of phosphatidic acid were determined by an ELISA, and therefore, we are unable to give the exact composition of the detected phosphatidic acid. Thus, in the following experiments, we used phosphatidic acid purified from egg yolk lecithin. We used a concentration of 5 μM phosphatidic acid in all cell culture experiments in the present study. To mimic stress in neurons, we treated PC-12 cells with 0.5 μg/mL corticosterone. Corticosterone-induced stress is a well-established, well-documented, and frequently reported model of stress for inducing depression-like symptoms in mice in vivo [8, 20, 23, 24, 35, 38, 42, 48, 56, 58]. Mice treated with corticosterone demonstrated symptoms of depression, for instance, decreased neurogenesis and depression-like symptoms in behavioral tests, and these symptoms are reversed by chronic treatment with antidepressants [8, 20, 35, 38, 42, 56]. According to these criteria, corticosterone treatment is a valid model of a depression-like state in mice. It is certainly always difficult to transfer in vivo data to a cellular system with much less complexity, but we have previously shown that treatment of neurons with corticosterone in vitro mimics at least some aspects of major depression that are seen in the brain of mice in vivo [21]. Thus, treatment of neurons with corticosterone seems be sufficient to study at least some aspects of cellular alterations in the pathogenesis of MDD.

We then determined the effects of phosphatidic acid in corticosterone-treated and untreated cells on typical regulators of autophagy, in particular the phosphorylation status of Ulk, Beclin, and Vps34. Autophagy has been previously shown to be inhibited in hippocampus neurons of mice treated by corticosterone or unpredictable environmental stress (21). The results showed that corticosterone increased the inhibitory phosphorylation of Ulk at the inhibitory serine 757 approximately twofold and decreased the activating phosphorylation of Ulk at serine 555 compared to untreated cells by approximately 60% (Fig. 1B, C). In accordance, corticosterone treatment reduced phosphorylation of Beclin and Vps34 and expression of p62 upon application by approximately 50% each (Fig. 1D–F). We next tested whether treatment of stressed cells by phosphatidic acid reverses the inhibition of Ulk, Beclin, and p62—measured as the phosphorylation and expression of the proteins. The data showed that treatment of the cells with phosphatidic acid reversed the effects of corticosterone on the phosphorylation and expression, respectively, of these proteins. Phosphatidic acid even increased the activating phosphorylation of Ulk, Beclin, and Vps 34 as well as expression of p62 in untreated cells over the levels observed in completely untreated cells (Fig. 1B–F).

**Fig. 2:**
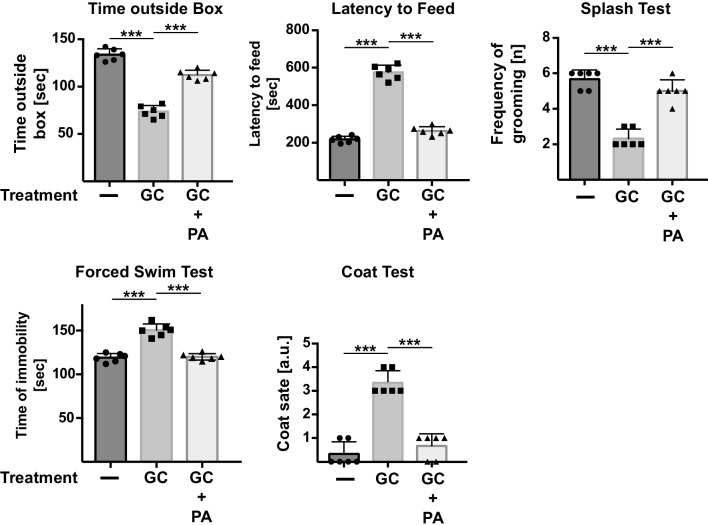
Phosphatidic acid restores normal behavior in mice with symptoms of major depressive disorder. Mice were treated with corticosterone (GC) for 6 days to induce experimental MDD. Phosphatidic acid (PA) was i.v. injected 24 and 12 h before analysis (i.e., twice at day 5) after initiation of the stress. Behavior was determined by using the time outside box, the latency to feed, the splash, the swim, and the coat test. Displayed are the mean ± SD from each of the 6 animals, ***p < 0.001, ANOVA and post hoc Tukey test

Next, we measured whether phosphatidic acid also impacts autophagy in vivo in corticosterone-stressed mice that served as model for MDD as described above. Corticosterone was applied for 5 days over the drinking water. We first tested whether intravenous injection of 4 μg/g body weight phosphatidic acid improves the behavior of mice stressed with corticosterone. The behavioral tests are described in detail in the methods; however, the time outside box and the latency to feed test mainly determine anxiety, while the coat state and the splash test determine self-care and self-interest. The swim test is an often-used assay to determine the effects of antidepressants and is a paradigmatic test for depressed behavior [62]. The results of these studies indicated that phosphatidic acid reversed the behavioral changes indicative of anxiety and depression/stress and induced by corticosterone (Fig. 2), confirming previous data [52].

**Fig. 3:**
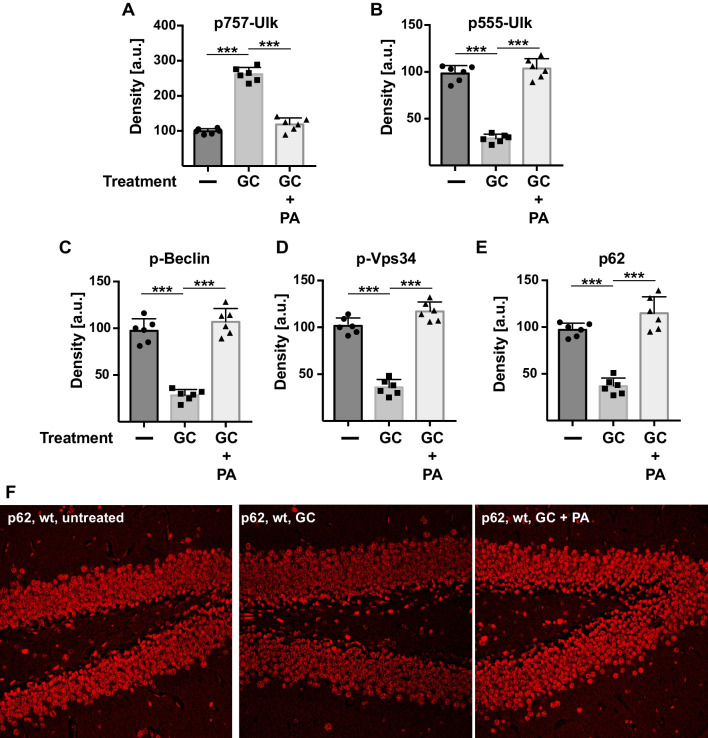
Phosphatidic acid prevents stress-induced inhibition of autophagy in vivo. The hippocampus was removed from mice that were left untreated, treated with corticosterone in the drinking water, or treated with corticosterone + i.v. injection of phosphatidic acid (PA). The hippocampus was homogenized by sonication in water, 5 × SDS-sample buffer was added, and proteins were separated by SDS-PAGE. Corticosterone (GC)-induced stress triggered inhibitory phosphorylation of Ulk at inhibitory serine 757 (**A**) and a concomitant dephosphorylation of Ulk at activating serine 555 (**B**), Beclin (**C**), and PI3-K/Vps34 (**D**); and a decrease in the expression of p62 in the hippocampus (**E**, **F**). Intravenous injection of phosphatidic acid 24 and 12 h prior to analysis reversed stress-induced changes and normalized phosphorylation and expression, respectively, of Ulk, Beclin, PI3-K/Vps34, and p62. Panels (**A–E**) show the quantitative analysis of western blots measuring the density of the protein bands. Panel (**F**) displays an immunostaining of the hippocampus performed with Cy3-coupled anti-p62 antibodies. Shown are the mean ± SD or representative example from each of the 6 animals, *p < 0.001, ANOVA and post hoc Tukey test

Next, we tested whether these behavioral changes also correlate with the activity of proteins regulating autophagy in the hippocampus. This was performed by measuring the phosphorylation status of Ulk, Beclin, and Vps3 as well as the expression of p62 in hippocampus extracts from mice left untreated, stressed with corticosterone, or treated with phosphatidic acid in the presence of corticosterone. These studies showed that corticosterone reduced the activating phosphorylation of Ulk, Beclin, and Vps34 and the expression of p62 in the hippocampus in vivo, effects that were reversed by i.v. injection of phosphatidic acid. Specifically, phosphatidic acid normalized the phosphorylation of Ulk, Beclin, and Vps34 and the expression of p62, respectively, in stressed mice to the levels observed in unstressed mice (Fig. 3A–E). Histological studies measuring the expression of p62 in the dentate gyrus of the hippocampus confirmed the downregulation of p62 in neurons of stressed mice and the normalization of p62-expression upon application of phosphatidic acid (Fig. 3F).

**Fig. 4:**
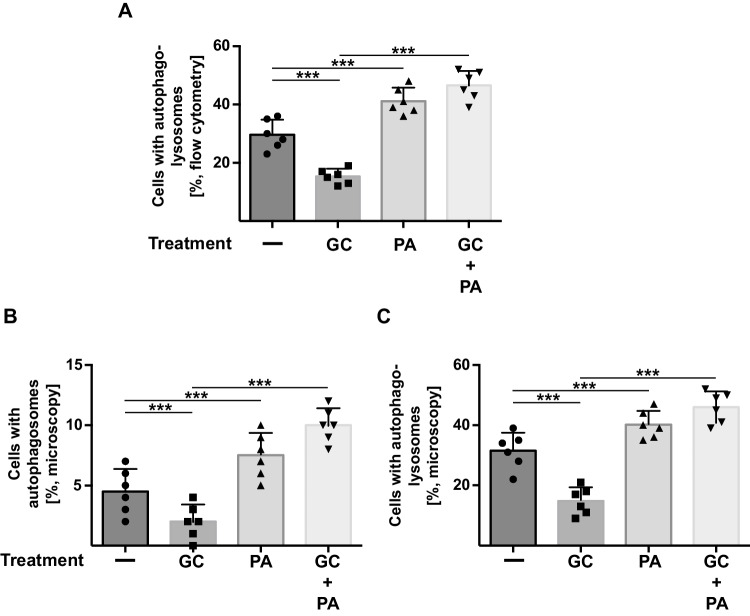
Phosphatidic induces autophagy in corticosterone-treated PC-12 cells. (**A**) To directly show the impact of phosphatidic acid on the fusion of autophagosomes with lysosomes, we transfected PC-12 cells with a RFP-GFP-Lc3B tandem construct, treated them with corticosterone (GC) in the presence or absence of phosphatidic acid or left the cells untreated, or treated the cells with phosphatidic acid (PA) only. Phagolysosomal fusion was determined by flow cytometry measurements of the proportion of GFP-low/RFP-high vs. GFP-high/RFP-high cells because the low pH of lysosomes quenches the GFP signal. Shown is the quantitative analysis of cells with autophagolysosomes from 6 independent studies; mean ± SD; ***p < 0.001, ANOVA and post hoc Tukey test. (**B**, **C**) To demonstrate the formation of autophagosomes or autophagolysosomes, respectively, PC-12 cells were transfected with a RFP-p62 (**B**) or a RFP-Lc3B (**C**) construct. The formation of autophagosomes/autophagolysosomes is indicated by large punctae in the cells. Cells were treated as in panel (**A**) and analyzed by fluorescence microscopy. Punctae were counted in 50 cells per sample and in 6 independent experiments (total of 300 cells). Given is the mean ± SD of the number of dots positive for p62 or Lc3B per cell; ***p < 0.001, ANOVA and post hoc Tukey test

To prove that phosphatidic acid directly regulates autophagy, we transfected PC-12 cells with a red fluorescent protein-green fluorescent protein-light chain 3β (RFP-GFP-Lc3B) tandem construct. Lc3B is incorporated into autophagosomes, which then fuse with lysosomes to autophagolysosomes. The labeling of Lc3B with GFP and RFP allows to determine the fusion of autophagosomes with lysosomes, which is indicated by reduction of the pH-sensitive fluorescence of GFP in acidic autophagolysosomes, while the fluorescence of RFP is not affected by the pH. We treated the cells with corticosterone in the presence or absence of phosphatidic acid and analyzed the samples by flow cytometry and determined the GFP signal in RFP positive cells. These flow cytometry studies demonstrated a reduction of autophagy upon incubation of PC-12 cells with corticosterone (Fig. 4A), consistent with previous data [19]. Co-incubation of corticosterone-treated PC-12 cells with phosphatidic acid restored autophagy (Fig. 4A). To confirm the notion that phosphatidic acid induces autophagy in stressed cells, we transfected PC-12 cells with an RFP-p62 (Fig. 4B) or an RFP-Lc3B (Fig. 4C) expression construct and determined RFP-positive punctae indicative for the formation of autophagosomes and autophagolysosomes, respectively, by microscopy analysis. The studies revealed that treatment of the neurons with corticosterone reduced the number of autophagosomes (Fig. 4B) and autophagolysosomes (Fig. 4C). Co-treatment with phosphatidic acid normalized the number of autophagosomes and autophagolysosomes (Fig. 4B, C).

Collectively, these studies demonstrated that phosphatidic acid induces and normalizes autophagy in corticosterone-stressed PC-12 cells.

Next, we aimed to identify targets in the hippocampus that are regulated by phosphatidic acid. A study by Cortassa and Maccioni indicated that phospholipids regulate the synthesis of gangliosides [6]. Thus, we hypothesized that the ganglioside GM1 might be a potential target of phosphatidic acid and we therefore determined the concentration of ganglioside GM1 in the hippocampus of mice that were treated with corticosterone or chronic unpredictable stress, or stressed and i.v. injected with phosphatidic acid. Chronic unpredictable stress, which cannot be changed or anticipated by the mouse, reflects another paradigm for the induction of MDD [36].

**Fig. 5:**
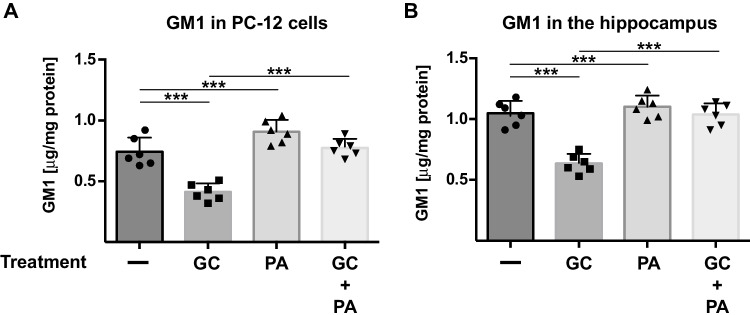
Phosphatidic acid induces GM1 in vitro and in vivo upon treatment of mice with corticosterone, (**A**) PC-12 cells were labeled with 1 μCi/mL [^14^C]galactose for 48 h. The cells were then either cultured with DMEM medium/0.1% fetal calf serum (untreated), stressed with corticosterone (GC), or treated with corticosterone + 5 μM phosphatidic acid (PA) for 3 h. We then pelleted the cells, extracted the cells in CHCl_3_:CH_3_OH:H_2_O (1:2:0.8, v/v/v), separated the phases by 5-min centrifugation at 14,000 rpm, and collected and dried the lower phase. Gangliosides were separated on TLC silica 60 plates using CHCl_3_:CH_3_OH:0.22%CaCl_2_ (60:35:8, v/v/v) and quantified employing a Fuji-Imager. GM1 was identified by co-migration with a standard. Shown are the mean ± SD of 6 independent experiments, *p < 0.001, ANOVA and post hoc Tukey test. (**B**) The hippocampus was removed from mice that were left untreated, treated with corticosterone (GC) in the presence or absence of i.v. phosphatidic acid (PA), or treated with PA alone. The hippocampus was homogenized by sonication in water, extracted in CHCl_3_:CH_3_OH:H_2_O (1:2:0.8, v/v/v), and lipids were analyzed. The TLC plates were sprayed with orcinol reagent, dried, developed at 120 °C for 30 min, and analyzed by densitometry. GM1 was quantified by comparison with a standard curve of known GM1 amounts. Protein was determined by a modified Bradford assay. Shown are the mean ± SD from each of the 6 animals, *p < 0.001, ANOVA and post hoc Tukey test

The results showed that both corticosterone and chronic unpredictable stress reduced GM1 levels in the hippocampus (Fig. 5). Intravenous injection of phosphatidic acid normalized the levels of GM1 in both forms of experimental MDD of mice (Fig. 5).

**Fig. 6:**
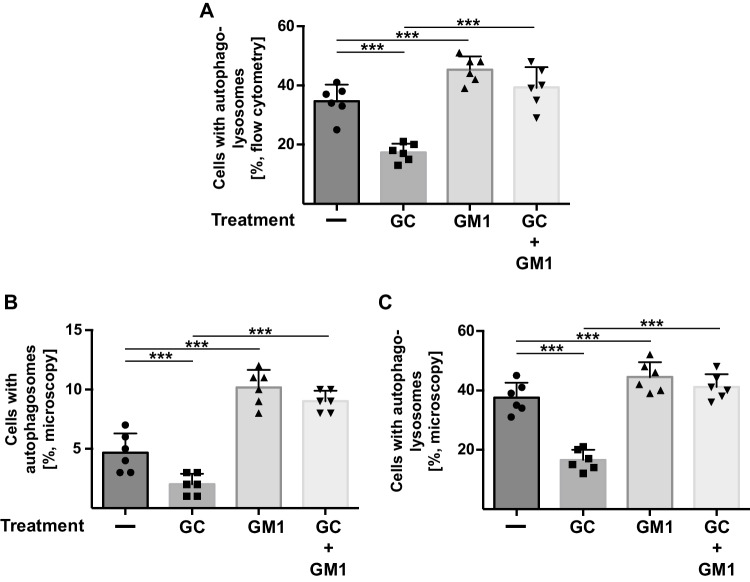
GM1 promotes autophagy in corticosterone-treated PC-12 cells. (**A**) PC-12 cells were transfected with a RFP-GFP-Lc3B tandem construct, treated with corticosterone (GC), treated with corticosterone + GM1, incubated with GM1 alone, or left untreated. Phagolysosomal fusion was determined by flow cytometry measurements as above. Given is the quantitative analysis of cells with autophagolysosomes from 6 independent studies; mean ± SD; ***p < 0.001, ANOVA and post hoc Tukey test. (**B**, **C**) Formation of autophagosomes after treatment of PC-12 cells with corticosterone-stressed ± GM1 was determined by transfection with expression constructs for RFP-p62 (**B**) or a RFP-Lc3B (**C**). The formation of autophagosomes/autophagolysosomes was indicated by large punctae in the cells, which were counted in 50 cells per sample and in 6 independent experiments (total of 300 cells) by fluorescence microscopy. Shown is the mean ± SD of the number of dots positive for RFP-p62 or RFP-Lc3B per cell; ***p < 0.001, ANOVA and post hoc Tukey test

To test whether the induction of GM1 by phosphatidic acid also relates to autophagy, we transfected PC-12 cells with the red fluorescent protein–green fluorescent protein–light chain 3 β (RFP-GFP-Lc3B) tandem construct (Fig. 6A), RFP-p62 (Fig. 6B), or an RFP-Lc3B (Fig. 6C) construct. We then treated the cells with corticosterone in the presence or absence of exogenous GM1 and determined the formation of autophagosomes and autophagolysosomes as above. The results showed that corticosterone suppressed the formation of autophagosomes and autophagolysosomes (Fig. 6A–C). Application of 10 μM GM1 restored the formation of autophagosomes and autophagolysosomes in PC-12 cells treated with corticosterone (Fig. 6A–C).

These studies established that corticosterone and chronic unpredictable stress reduce GM1 levels in the hippocampus. In vitro, GM1 restored autophagy in corticosterone-treated cells.

**Fig. 7:**
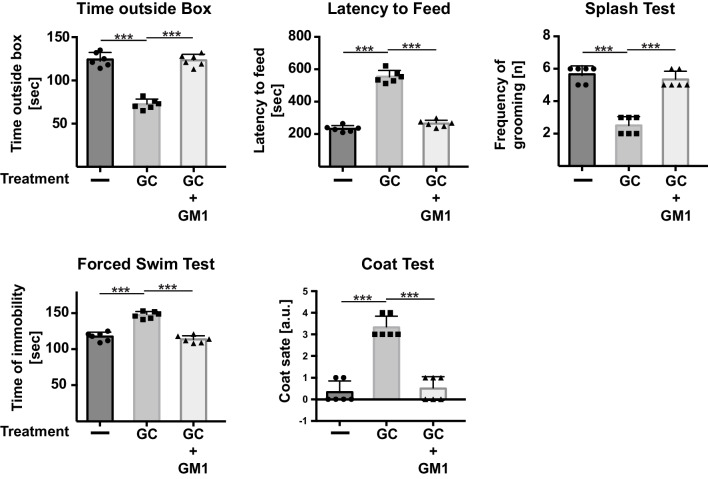
Intravenous injection of GM1 prevents experimental major depressive disorder in stressed mice. Mice were treated with corticosterone (GC) or chronic unpredictable environmental stress (CUS) for a total of 6 days. GM1 was i.v. injected twice at day 5, i.e., 24 and 12 h prior to analysis. Behavior was determined at day 6 after stress initiation. Displayed are the mean ± SD from each of the 6 animals, *p < 0.001, ANOVA and post hoc Tukey test

These studies raise the question whether treatment of mice with GM1 is able to influence corticosterone-induced behavioral changes in mice. To address this question, we determined whether repeated intravenous injection of GM1, which has been shown to reach the brain [12], had an impact on the behavior of corticosterone-stressed mice. The results showed that injection of GM1 into mice that were treated with corticosterone reduced anxiety in the time outside the box and latency to feed tests and reversed depressive behavior in the splash, coat, and swim tests (Fig. 7).

## Discussion

Several studies have shown that ceramide is increased in the blood plasma of patients with major depressive disorder [4, 33, 52]. We have shown that this increase of ceramide also causes symptoms of experimental MDD in mouse models and that neutralization or degradation of blood plasma ceramide prevents stress-induced MDD-like symptoms in mice [52]. We also demonstrated that blood plasma ceramide results in inhibition of phospholipase D in endothelial cells of the brain [52]. We further demonstrated that inhibition of phospholipase D in mice induces behavioral changes similar to those in MDD, while, vice versa, the i.v. injection of phosphatidic acid into mice treated with corticosterone or chronic environmental stress rapidly corrected depressed behavior in the animals. These studies suggested that a reduction of phosphatidic acid, for instance, in the hippocampus, mediates the effects of increased blood plasma ceramide on major depression [52].

Here, we aimed to identify molecular mechanisms how phosphatidic acid acts against MDD. We treated cells and mice with corticosterone, which is a well-accepted stress stimulus in mice and often used in experimental studies on MDD [8, 20, 23, 24, 35, 38, 42, 48, 56, 58]. Cells incubated with corticosterone were then treated with phosphatidic acid at a concentration that is similar to the concentration in blood plasma. Mice were i.v. injected with a higher dose of phosphatidic acid since (i) the lipid will certainly also interact with other cells in the blood and not only with endothelial cells and (ii) only a small fraction of phosphatidic acid will be able to pass the blood–brain barrier and reach neuronal cells. These studies demonstrated that phosphatidic acid regulates autophagy in cultured PC-12 cells, but more importantly also in the hippocampus in vivo. We have previously shown that corticosterone treatment and chronic unpredictable environmental stress reduce autophagy in the hippocampus of mice and that inhibition of autophagy induces symptoms similar to MDD in these mouse models of experimental MDD [21]. In addition, it was shown that anti-depressants such as amitriptyline and citalopram induced autophagy in rat primary neurons [66]. Autophagy is also stimulated by lithium that is often used to treat patients with MDD [28], by ketamine [65], electroconvulsive therapy [41], and physical activity [26], all treatment modalities against MDD. Thus, reduced autophagy might be an important pathogenetic factor in MDD and its correction via different pathways may finally have the same result, i.e., improvement of major depressive disorder. However, it should be indicated that it is unknown whether induction of autophagy improves behavior in other experimental animal models with MDD-like symptoms. It is also unknown whether autophagy is altered in patients with MDD and whether antidepressants also trigger autophagy in humans. Our data link phosphatidic acid with the regulation of autophagy. The data suggest that increased blood plasma ceramide levels mediate a downregulation of autophagy by decreasing phosphatidic acid concentrations in the hippocampus, which results in symptoms of MDD. In this model, antidepressants act in neurons to improve autophagy [21, 23] and thereby counterbalance the effects of reduced phosphatidic levels in the hippocampus.

Our data further suggest that phosphatidic acid regulates the synthesis of the ganglioside GM1 in vitro and in vivo. GM1 has been previously linked to the induction of autophagy [7] and our studies support the notion that GM1 or GM1 metabolites regulate autophagy since the reduced autophagy in corticosterone-treated neurons is corrected by the addition of GM1. Thus, it might be possible that reduced concentrations of phosphatidic acid in the hippocampus of stressed mice result in reduced formation of GM1 and thereby, most likely indirectly, in a reduction of autophagy. The significance of decreased GM1 levels for the pathogenesis of MDD is indicated by *the *in vivo studies demonstrating that i.v. injection of GM1, which has been shown to pass the blood–brain barrier [12], corrects depression-like behavior in mice with experimental MDD. However, it is unknown whether GM1 is changed in hippocampus neurons of patients with MDD.

Our studies only show a correlation between the effects of phosphatidic acid and GM1 on autophagy and depressive behavior. Thus, they do not exclude additional effects of phosphatidic acid and GM1 in the regulation of neuronal functions. These pathways need to be identified in future studies and their characterization seems to be beyond the present manuscript. At present, it is also unknown how phosphatidic acid regulates the formation of GM1 and how GM1 regulates autophagy.

It is unknown whether phosphatidic acid and GM1 are altered in the brain of human patients with MDD. It seems to be impossible to obtain valid information on these lipids in the patients since lipids might be rapidly altered in postmortem brains. At present, no methods are available to visualize these lipids in patients in vivo.

Phosphatidic acid has been previously shown to be involved in the regulation of autophagy. Thus, studies on hepatocytes demonstrated that deletion of phospholipase D1 blocked the autophagic flux in these cells in addition to mitochondrial alterations [34]. Addition of phosphatidic acid restored the dynamics of autophagy, consistent with the present study. Likewise, overexpression of PLD1 in hepatocytes induced autophagy [54]. Phosphatidic acid seems to drive autophagy by biophysical and biochemical mechanisms. Thus, it was shown that ATG3 strongly interacts with negatively charged phospholipids, in particular cone-shaped negatively charged phospholipids such as phosphatidic acid [31]. ATG3 promotes the formation of autophagosomes by triggering the binding of LC3 to lipid membranes and has been also proposed to facilitate LC3/GABARAP lipidation, thereby driving autophagy [26]. Furthermore, phospholipase D2 and phosphatidic acid have been demonstrated to activate Akt [5]. Akt in turn phosphorylates Beclin1, which promotes autophagic flux [5], consistent with the present data.

On the other hand, phosphatidic acid has been shown to activate mTOR in response to amino acids and nutrients, which blocks autophagy [17].

Thus, it might be possible that phosphatidic acid mainly functions as a co-stimulus that depends on the context with other stimuli.

In summary, our data demonstrate that phosphatidic acid promotes autophagy in vitro in stressed neurons and in vivo in the hippocampus of stressed mice. The effects of phosphatidic acid seem to be mediated, at least in part via a regulation of GM1 levels, which are reduced by stress and upregulated by phosphatidic acid.

## Data Availability

All data are presented in the manuscript.
